# Digestibility of Meat Mineral and Proteins from Broilers Fed with Graded Levels of *Chlorella vulgaris*

**DOI:** 10.3390/foods11091345

**Published:** 2022-05-05

**Authors:** Marija Boskovic Cabrol, Joana C. Martins, Leonardo P. Malhão, Cristina M. Alfaia, José A. M. Prates, André M. Almeida, Madalena Lordelo, Anabela Raymundo

**Affiliations:** 1 LEAF—Linking Landscape, Environment, Agriculture and Food, Instituto Superior de Agronomia, Universidade de Lisboa, Tapada da Ajuda, 1349-017 Lisboa, Portugal; ju.martins059@gmail.com (J.C.M.); leonardomalhao@gmail.com (L.P.M.); aalmeida@isa.ulisboa.pt (A.M.A.); mlordelo@isa.ulisboa.pt (M.L.); anabraymundo@isa.ulisboa.pt (A.R.); 2 Department of Food Hygiene and Technology, Faculty of Veterinary Medicine, University of Belgrade, 11000 Belgrade, Serbia; 3 CIISA—Centro Interdisciplinar de Investigação em Sanidade Animal, Faculdade de Medicina Veterinária, Universidade de Lisboa, Avenida da Universidade Técnica, Alto da Ajuda, 1300-477 Lisboa, Portugal; cpmateus@fmv.ulisboa.pt (C.M.A.); japrates@fmv.ulisboa.pt (J.A.M.P.)

**Keywords:** poultry, pectoralis major, microalgae, amino acids, protein recovery, mineral bioaccessibility

## Abstract

The incorporation of sustainable protein sources in animal feeding is a growing trend. So far, no study has investigated in vitro digestion of meat, from broilers fed microalgae, in a human model. This research aimed to evaluate the effect of incorporating *Chlorella vulgaris* in the broilers diet on human protein digestibility, and mineral bioaccessibility. The study used 240 male Ross 308 broilers randomly allocated to groups fed a control diet or a diet where soybean meal was replaced with 10% (CV10%), 15% (CV15%), or 20% (CV15%) of *C. vulgaris* for 40 days. The microalga supplementation increased the protein and lowered the fat content in the muscle. Results on the percentages of amino acids highlighted that arginine and threonine proportions increased and lysine and cysteine proportions decreased with microalga inclusion. CV15% and CV20% meat had higher amount of K, Ca, Mg, P, and Fe in raw breasts, improving the nutrient composition of the meat. Cooking caused a decrease in Na and K and an increase in other minerals. CV20% had higher bioaccessibility of K, Ca, Mg, P, and Mg, compared to the control. Replacing soybean meal in broiler feed with higher concentrations of *C. vugaris* could improve the digestibility of meat protein and minerals.

## 1. Introduction

Poultry meat is one of the most consumed meats worldwide, mainly due to the affordable price and the perceived healthy nutritional profile by comparison to red meat [1]. Chicken meat is the source of high-value proteins, minerals, and vitamins [2]. Moreover, poultry meat consumption as part of a well-balanced diet is linked to a lower risk of certain diseases, including obesity, cardiovascular diseases or type 2 diabetes mellitus [3]. Broiler production is mostly conducted in intensive farming systems using imported feedstuffs such as corn, other grains, and soybean meal. The transport costs of such feedstuffs, mostly produced in the Americas and Black Sea regions, raise sustainability concerns. As such, novel alternative feedstuffs are required in order to address such challenges and increase the sustainability of production systems. Additionally, diet-related health concerns also raise interesting opportunities by increasing meat nutritional value by dietary modification.

One of the promising opportunities for reducing the environmental impacts of livestock production systems is replacing major feedstuffs, such as soybean meal, with alternative protein sources [4]. Microalgae, including *Chlorella vulgaris*, were previously reported to be a sustainable feed source of proteins with much higher photosynthetic capacity than higher plants [5]. The cultivation of microalgae is considered as an eco-friendly process [6]. Indeed, microalgae biomass production requires simple conditions (CO_2_ and sunlight), a small area of non-arable land, and furthermore, it is not affected by seasonality. As other advantages, microalgae cultivation does not require pesticides as in conventional crops do and microalgae production provides higher yields [6,7,8]. Research evidence has shown that partial replacement of soybean meal with microalgae, including *C. vulgaris*, in broiler feed enriches meat with PUFA (in particular eicosapentaenoic acid (EPA) and docosahexaenoic acid (DHA)), as well as pigments such as chlorophylls and carotenoids [9,10,11,12]. Only a few studies provide evidence of *C. vulgaris* influence on the amino acid composition of meat [10], while, to the best of our knowledge, no information on its effect on mineral content is available. To evaluate the nutrient content available for human nutritional use, the determination of the total nutrients in food does not provide sufficient information. Thus, it is important to establish the amount of nutrients that are potentially accessible after digestion [13]. Bioaccessibility refers to the fraction of the nutrient that is released from the food matrix that is thus soluble in the gastrointestinal environment and available for absorption [14]. When determining bioaccessibility, it is important to consider the way food is being consumed, since the thermal process can influence the concentration of accessible nutrients, mainly due to cooking losses [15]. To the best of our knowledge, there is no data on the digestibility and mineral bioaccessibility of meat from broiler chickens fed microalgae.

This study aimed to assess the nutritional value of the breast meat of broilers fed different levels of *C. vulgaris* for human nutrition. For this purpose, chemical and amino acid composition, protein digestibility, and bioaccessibility of minerals, by in vitro simulation of gastrointestinal digestion using a standardized semi-dynamic INFOGEST model [16], were determined.

## 2. Materials and Methods

### 2.1. Animal Housing and Experimental Diets

The study was conducted at the research facilities of Instituto Superior de Agronomia (ISA), University of Lisbon (Portugal). Animal experimentation procedures were approved by the Animal Welfare Research and Ethics Commission (ORBEA) of ISA. All appropriate legislation and guidelines on animal experimentation of both Portugal and the European Union were followed. Two hundred and forty, one-day old male Ross 308 broilers were submitted to an adaptation period of 4 days, and a corn/soybean meal standard diet was provided during this period. At 5 days of age, broilers were randomly allocated into four groups, each with 60 animals (six replicate pens per treatment with 10 birds per pen). The control group (C) was fed a standard diet without the inclusion of *C. vulgaris*. In the experimental groups, *C. vulgaris* (Table 1) was incorporated in the diet at concentrations of 10 (CV10%), 15 (CV15%), and 20% (CV20%), respectively. Clean water and feed were provided ad libitum throughout 40 days and the floor of the pen consisted of wood shavings. A starter diet was fed from day 5 to 18 (phase I) and a grower diet was fed from day 19 to 40 (phase II). All diets were formulated to contain adequate nutrient levels for the broiler Ross 308 strain at different ages as summarized in Table 2, while amino acid composition of the feed is presented in Table 3. Room temperature was set according to the requirements of the breed at each specific age. Animal performance parameters (body weight, feed consumption, average daily gain and carcass yield, among others) were recorded and are described in a companion paper (submitted results).

### 2.2. Animal Slaughtering and Sampling

At the end of the experiment (day 40), 3 broilers per pen (18 broilers per group) were transported to a commercial poultry slaughterhouse, electrically stunned, and immediately slaughtered by severance of the jugular veins according to standard commercial practices in the European Union. During the first 24 h post mortem, carcasses were stored in a ventilated cold room until reaching 4 °C. Then, fresh pectoralis major muscle was removed, vacuum packed and stored at −20 °C until further analysis.

### 2.3. Proximate Chemical Composition, Cholesterol Level and Energy Value

For the chemical composition, 5 g of breast meat was trimmed of visible adipose and connective tissue and homogenized. Moisture was determined by drying samples for 24 h at 105 °C, until constant weight. After moisture determination, and for ash determination, dried samples were incinerated at 550 °C in a muffle until constant weight (approx. 16 h). The fat content was determined by the Soxhlet method [17], using petroleum ether as solvent. To determine protein content, 0.2 mg of fresh and cooked sample (sous vide at 80 °C to reach a core temperature of 77 °C) were weighed in a tin foil and analyzed using the Dumas method and a Thermo Quest NA 2100 Nitrogen and Protein Analyser (Interscience, Breda, The Netherlands), using a nitrogen-to-protein conversion factor of 6.25. Each analysis was performed in triplicate, for six breasts per group. Total cholesterol was determined according to Prates et al. [18]. After direct saponification with solution composed by potassium hydroxide, ethanol, and deionized distilled water, breast meat samples (0.75 g) were incubated in a shaking water bath at 80 °C for 15 min. Then, samples were centrifuged at 2500 rpm for 10 min, and an aliquot of the n-hexane layer was filtered and injected into an HPLC system (Agilent 1100 Series, Agilent Technologies Inc., Palo Alto, CA, USA), using a normal-phase silica column (Zorbax RX-Sil, 250 mm × 4.6 mm i.d., 5 μm particle size, Agilent Technologies Inc., Palo Alto, CA, USA) with UV-visible photodiode array detection of cholesterol (λ = 202 nm) in series. Total cholesterol was determined based on the external standard method from a standard curve of peak area vs. concentration. Energetic value was calculated using the relative percentage of protein and fat which was multiplied by the correction factors, 4 and 9 kcal/g, respectively, according to the Regulation (EU) No 1169/2011 [19].

### 2.4. Total Amino Acid Profile

The amino acid profile of the chicken breast meat was analyzed according to the methods described by Tian et al. [20]. One gram of Pectoralis major muscle was homogenized with 10 mL HCl (6 mol/L) in hydrolytic tubes for 24 h at 110 °C. Then, hydrolysates were diluted with 0.02 mol/L HCl in 50 mL volumetric flasks and mixed. One ml of solution was evaporated to dryness in a water bath at 65 °C, dissolved in 2 mL HCL, and filtered through filter paper. Then, amino acids were derivatized by online column derivatization using o-phthalaldehyde for primary amino acids and 9-fluorenylmethyl chloroformate for secondary amino acids. The derivatized amino acids were analyzed by reversed-phase HPLC (Agilent 1100, Agilent Technologies, Avondale, PA, USA), using a column (Gemini C18 column, 150 × 4.60 mm, 5 µm; Phenomenex, Torrance, CA, USA). The separation was performed at 40 °C using a gradient between 2 solvents: 40 mM sodium phosphate at pH 7.8 (solvent A) and acetonitrile: methanol–water (45:45:10 *v*/*v*, solvent B). The flow rate was 2 mL/min. The detection was monitored using the fluorescence signal monitored at 450 nm for emission and 340 nm for excitation.

### 2.5. Mineral Profiling

The concentration of Ca, Cu, Fe, K, Mg, Mn, Na, P, and Zn was determined in both raw and cooked meat and in the liquid digesta upon completion of in vitro gastrointestinal-simulated digestion. For mineral analysis, 0.5 g of homogenized raw and cooked breast and soluble fraction obtained after in vitro digestion, was digested on a MARS 240/50 microwave digester (CEM Corporation, Mathews, NC, USA), using 9 mL hydrochloric acid (HCl) and 3 mL ultra-pure nitric acid (HNO_3_), and cooled at room temperature. Deionized water was added to Falcon tubes up to 50 mL and inductively conducted plasma-optical emission spectrometry was conducted with iCAP 7000 Thermo Fisher ICP-OES (Thermo Fisher Scientific, Waltham, MA, USA). Results were corrected to account for the dilution factors resulting from the digestion procedure, and presented as mg/100 g breast meat on a wet-weight basis.

The bioaccessibility (BA) of minerals in broilers breast, expressed as percentages was calculated as follows:

BA% = BC/TC × 100(1)

where BC = bioaccessible concentration of minerals (released fraction in digestive fluid); TC = total concentration of minerals in sample before digestion, expressed as mg/100 g.

### 2.6. In Vitro Digestion

The INFOGEST protocol [16] was used in order to simulate the three phase in vitro gastrointestinal human digestion: oral, gastric and intestinal. Digestion experiments were carried out in triplicate. As for the enzyme-blank, a tube containing 2 mL of ultrapure water (Milli-Q^®^) without sample was used. Electrolyte stock solutions (SFS, SGF, SIF) were prepared according to Brodkorb et al. [16].

In the oral phase, 2 g of cooked minced breast meat was added to falcon tubes and mixed with 1.6 mL of simulated salivary fluid electrolyte stock solution, 0.03 mL CaCl_2_(H_2_O)_2_ (0.1 M) and 0.37 mL of deionized water comprising 150 U/mL of porcine α-amylase. The mix was kept at 37 °C for 2 min under agitation. Following the oral phase, samples were mixed with 3.2 mL of gastric fluid electrolyte stock solution and pH of the mix was adjusted to 3 with 1 M HCl solution. Then, 0.006 mL of 0.1 M CaCl_2_, ultrapure water (to achieve a total volume of 4 mL) and pepsin were added (4000 U/mL). The mix was kept at 37 °C for 2 h with a constant mixing (30 rpm). Finally, 6.4 mL of simulated intestinal fluid was added to the sample and the pH of the reaction solution was immediately adjusted to 7.0 with a 1 M NaOH solution followed by addition of freshly prepared bile solution to reach a final concentration of 10 mM, 0.048 mL CaCl_2_(H_2_O)_2_ (0.1 M), ultrapure Milli-Q water (to achieve a total volume of 8 mL), pancreatine (200 U/mL based on trypsin activity) and incubated at 37 °C for 2 h. Once the intestinal phase was finished, enzymes were inactivated by adding Pefabloc^®^. After inactivation, samples were centrifuged at 10,000 rpm for 10 min at 4 °C and separated into supernatant (soluble part/the fraction available for absorption) and residue (insoluble part). Supernatants were stored at −80 °C until further analysis.

### 2.7. Protein’s Recovery after In Vitro Digestion and Digestibility

The protein concentration of samples after in vitro digestion was determined by the Dumas method (Thermo Quest NA 2100 Nitrogen and Protein Analyser, Interscience, Breda, the Netherlands), using a nitrogen-to-protein conversion factor of 6.25. All supernatant and pellets after in vitro digestion were measured by weighing 250 mg of sample into tin foils. The recovery of proteins was calculated per tube as described previously [21]. Briefly, the amount of total dissolved protein of each sample was calculated by multiplying protein concentration (obtained by combustion analysis) with the total liquid volume of each sample (including sample moisture content, the volume of simulated fluids and HCL and NaCl used for pH adjustment) and subtracting the protein contribution of the enzyme blank. The insoluble protein fraction amount was calculated based on the weight of residue and its protein content. Then, soluble protein fraction (SPF), insoluble protein fraction (IPF), and protein recovery (PR) contents were calculated and expressed as follows:

SPF (%) = (Protein in soluble fraction (mg) × 100)/Protein in the sample before digestion (mg)(2)

IPF (%) = (Protein in residue (mg) × 100)/Protein in the sample before digestion (mg);

PR (%) = (Protein in soluble fraction (mg) + protein in residue (mg) × 100)/Protein in the sample before digestion (mg).

Digestibility was calculated as follows:

Digestibility (%) = (W0 − W1)/W0 × 100(3)

where W1 is protein content (mg) in the precipitate after digestion and W0 is protein content (mg) in the untreated product before digestion.

### 2.8. Statistical Analysis

Statistical analyses were performed using the software GraphPad Prism version 9.00 for Windows (GraphPad Software, San Diego, CA, USA, www.graphpad.com). The effects of different dietary treatments were appraised by one-factor variance analysis (ANOVA) followed by Tukey’s multiple comparison tests. Significance was declared when *p* < 0.05.

## 3. Results and Discussion

Growth and carcass traits were submitted in detail in a companion paper (unpublished data). The results are briefly shown here for contextual reasons. We have demonstrated that the production performance was improved (*p* < 0.05) when 10% of soybean meal was replaced with *C. vulgaris*, in comparison to the remaining groups, but not different from the control (*p* > 0.05). Namely, at the end of experiment (day 40) the body weight, weight gain, and feed intake of the control and CV10% group were 2801 and 2819 g, 70.80 and 63.87 g/day, and 40,425 and 40,075 g/pen, respectively. Compared with the control group, birds supplemented with higher concentrations of *C. vulgaris* (15% and 20%) had lower body weight (2587 and 2342 g), weight gain (70.80 and 63.87 g/day), and feed intake (37,382 and 35,922 g/pen), while feed conversion ratio did not differ between control and CV groups.

From a nutritional point of view, the quality of meat, pigment concentrations, and antioxidant activity is dependent on the inclusion levels and increase with *C. vulgaris* inclusion. Furthermore, the addition of *C. vulgaris* in broilers feed was an efficient way to significantly increase the concentration of DHA+EPA and improve the n-6/n-3 ratio in broilers breast meat without affecting texture and sensory acceptance of the meat. The present study aimed to complement primary nutritional data with the effect of microalgae in broilers feed on mineral, amino acid composition, and digestibility of meat.

### 3.1. Chemical Composition, Cholesterol Level, and Energy Value

The effect of dietary microalgae on the proximate composition of breast meat is presented in Table 4. The incorporation of 15 and 20% *C. vulgaris* resulted in an increase of protein content in the meat (*p* < 0.05). Ash content was increased in all groups where this microalga was included, although no significant differences were found between them. Fat content was significantly lower (*p* < 0.05) in the breast muscle of broilers that were fed a diet with *C. vulgaris*, in comparison to those fed the control diet. No significant differences were found for moisture, cholesterol, and energy contents between groups.

It is evident from the results that meat from groups of broilers fed *C. vulgaris* was richer in protein. The contribution of proteins to the total energetic value of control, CV10%, CV15%, and CV20% breast was 76.11, 85.34, 92.90, and 90.73%, respectively. Such higher protein content could be related to the higher availability of amino acids originated from the *Chlorella* proteins, an improved absorption of nutrients, or both. Accordingly, Mirzaie et al. [22] reported that 10 g/kg dietary supplementation with *Chlorella* by-products significantly increased jejunum villus heights and crypt depths, increased absorption area, and consequently feed utilization. Furthermore, Kang et al. [23] and Janczyk et al. [24] found that *C. vulgaris* addition in feed significantly increased *Lactobacillus* spp. in the broiler’s intestines and caeca of laying hens. This is particularly relevant as beneficial microorganisms in the intestinal tract enhance digestion and nutrient absorption [23]. As such, and in agreement with our results, Kalbe et al. [25] found protein increase in pork from pigs fed Schizochytrium spp. Furthermore, it has been suggested that DHA supplementation stimulates muscle protein synthesis in growing pigs [26]. This agrees with findings from the present study, where DHA was significantly higher in experimental groups fed *C. vulgaris* (up to 16 times; submitted data). Additionally, Waldroup et al. [27] showed that greater amount of proteins and single amino acids in broiler feed resulted in elevated protein content of meat. As presented in Table 3, the diets with *C. vulgaris* inclusion resulted in higher crude proteins in broiler feed due to an increase in amino acids, mostly lysine and phenylalanine. Our results corroborate previous studies reporting a slight increase in crude proteins from broiler feed when microalgae were used as a protein source in high concentrations [9,28]. This change in feed proteins is due to differences in protein content of soybean meal and microalgae used. While crude protein of soybean meal in the present study was 43%, according to standardized soybean meal protein content on the feed market [29], *C. vulgaris* from the present study contained 46% of proteins (Table 1). The major determinant of protein deposition is the dietary supply of amino acids [30]. However, it was suggested that after achieving optimal feed requirements for crude protein and lysine, birds’ growth rates reach their maximum, and further supplementation only results in a plateau effect [31]. When feed contains equal to or more than 210 g/kg of crude proteins and 1.22% of lysine, further supplementation does not increase muscle protein deposition [32]. Even though the crude protein level in the present study was high, the lysine concentration was below 1.22%. Furthermore, it is worth mentioning that broiler hybrids have different growth requirements and genetic potential. Thus, we speculate that higher protein content in feed led to higher amino acid content available for absorption and de novo synthesis of muscle proteins within genetically predetermined broiler potential. Apart from the aforementioned, the elevated protein level can be partially attributed to the lower-fat participation in the relative weight of meat samples from broilers fed *C. vulgaris* compared to control.

Regarding fat content, the Food Advisory Committee [33] stated that meat containing less than 5% is considered to be “lean meat”. In the present study, fat content in the breast meat ranged from 0.92 to 3.40%. However, “low fat” health claims can be applied only to breast meat from broilers fed *C. vulgaris* groups that had a fat content lower than 3 g/100 g [19,34]. Corroborating our study, lower intramuscular fat in meat from pigs fed 2 g/d *Spirulina platensis* was reported by Šimkus et al. [35]. Namely, a lower amount of fat in the muscle is due to fatty acid modification of diets. Diets enriched with n-3 PUFA are associated with reduced fat deposition [36]. The microalgae, including *C. vulgaris*, are a source of n-3 PUFA [9]. In a companion paper with the same experimental design (data unpublished) the amount of n-3 PUFA in breast meat from broilers fed *C. vulgaris* increased between 2.6 and 5.4 times relative to control. In addition, De Tonnac et al. [37] suggested that the inclusion of DHA in the feed inhibited the expression of sterol regulatory element-binding protein 1, a transcription factor that regulates the expression of genes encoding lipogenic enzymes, and resulted in reduced lipid contents in pig muscle. As mentioned above DHA was significantly higher in meat from broilers fed microalgae, which could influence regulation of the same or similar proteins in birds.

The increase in ash content of the meat as a consequence of Chlorella inclusion is likely due to the greater amount of minerals in meat from the broilers fed *Chlorella* (Table 4) and is related to the mineral composition levels of the different diets. This subject will be addressed later in this Discussion.

Moisture and cholesterol content did not differ among breast meat from different dietary treatments (*p* > 0.05). Raw poultry meat has approximately 27 to 90 mg cholesterol/100 g [38,39]. In the present study, the cholesterol level from the breast meat was in that range and was not affected by dietary treatment. Such concur with results from previous studies using microalgae inclusion in broilers feed [9,11].

### 3.2. Amino Acid Profile

Although microalgae have gained a lot of attention as a source of sustainable feed protein, most studies are focused on their effect on fatty acid composition, whilst the amino acid profile of chicken meat has been scarcely investigated [10]. The influence of dietary *C. vulgaris* on breast meat amino acid profile is summarized in Table 5. Lysine, arginine, and leucine were the most representative amino acids among the essential amino acids, while glutamic acid, aspartic acid, and alanine were the most abundant among non-essential amino acids. Arginine and threonine proportions were significantly affected by the dietary treatment. The breast meat from groups fed *C. vulgaris* had significantly higher concentrations of these amino acids than those in the control group (*p* ˂ 0.05) in total amino acid content. In addition, meat from the CV15% and CV20% group had a lower content of lysine, compared to control, while the inclusion of *C. vulgaris* decreased cysteine participation of total amino acid profile. Other amino acids proportions of breast muscle did not differ among groups (*p* ˂ 0.05).

Despite being one of the most consumed types of meats, and nutritionally important for humans, there are only a few reports on the amino acid content and profile of chicken meat in the literature [40]. Findings from the present study for amino acid composition, are in accordance with results provided by other authors for broiler breast meat [10,40,41,42]. Such studies confirm that arginine, lysine, leucine, glutamic acid, and aspartic acid are the most abundant in broiler breast meat. Until now, only one study evaluated the effect of *C. vulgaris* supplementation on breast meat amino acid composition [10]. The authors did not report any difference in amino acid composition from meat-fed *C. vulgaris* and the control group. However, they included microalgae in a low amount (1 g/kg diet) in opposition to the present study where high concentrations of *C. vulgaris* were added to broilers feed.

### 3.3. Mineral Composition and Bioaccessibility

The macro- and micromineral contents for raw and cooked breast meat are presented in Table 6. The major minerals present in raw and cooked meats were macromineral K, followed by P, Na, and Mg. The levels of Ca, Fe, Cu, Zn, and Mn in the groups from different dietary treatments ranged from 4.69 to 6.66, 1.09 to 1.58, 0.053 to 0.065, 1.02 to 1.27, and 0.01 to 0.025 g/100 g in raw and from 4.83 to 7.22, 1.11 to 1.69, 0.13 to 0.15, 1.03 to 1.64, and 0.024 to 0.029 in cooked meat samples, respectively.

The literature data on chicken meat mineral composition are limited. Values found for the most of the measured minerals in the present study were in the ranges given by other authors for raw breast meat [43,44]. The K, Ca, Mg, P and Fe content of the breast were significantly increased by 15 and 20% dietary inclusion of *C. vulgaris* (*p* ˂ 0.05). It has been previously reported that the microalgae *C. vulgaris* is rich in minerals, in particular potassium, phosphorus, magnesium, and iron [45,46,47].

One of the most important findings of the present study is that microalgae inclusion in broilers diet enhanced iron content in breast meat. While red meat is the most important food source of iron in the diet, white meat is perceived as healthier than red meat by consumers [48], despite containing a significantly lower amount of this mineral. Iron deficiency is one of the most widespread nutritional problems resulting in different pathological conditions, including anemia [49]. The iron content in breast meat, like other minerals, was found to be strongly dependent on the *C. vulgaris* inclusion. In the present study addition of 10, 15, and 20% of *C. vulgaris* increased iron content by 8.7, 45.1, and 33.2% respectively, by comparison to controls, reaching an iron content similar to those of red meats. The addition of *C. vulgaris* also increased the phosphorus amount in breast meat. This increase is partially explained by *C. vulgaris* being a good source of phosphorus and in another part due to the chemical form of phosphorus found in microalgae. While the form of phosphorus found in plants is organic phytic acid, the phosphorus is stored in microalgae in the form of polyphosphate granules [50]. Thus, apart from *C. vulgaris* inclusion, other dietary factors in broiler feed should also be considered. In this regard, it is important to mention that soybean contains antinutritional factors including phytic acid and its salt, phytates. Phytate are heat-stable and cannot be eliminated by the heat treatment during the processing of soybean meal [51]. In addition, the concentration of phytates is lower in the hulls than in the cotyledons, which is why dehulling and oil extraction from whole soybean will lead to an increase in phytate concentration [52,53]. The phytate content ranges approximately from 1.4 to 1.6% [54] in soybean meal, and from 1.00 to 1.47% on a dry matter basis in soybean [55], respectively. Despite being represented in low amounts, phytic acid and phytate have the capacity to chelate positively charged cations, especially calcium, iron, zinc, and magnesium, and reduce their bioavailability in poultry [56]. Consequently, lower absorption of these elements could lead to their lower deposition in meat in the control group, where soybean meal content was higher between 10 and 20% compared to experimental groups. However, this explanation cannot apply to zinc, since contrarily to other minerals, zinc was lower in broiler meat by the addition of *C. vulgaris* in diet. Although not significant, the decrease in Zn in meat could be a result of its lower absorption due to an antagonism with iron or copper [57], which increased in meat from all groups fed *C. vulgaris* by 22.64%. The decrease of Zn content was also noted by Saeid et al. [57] in pork from pigs fed diets containing *Spirulina maxima* biomass enriched with Cu. In addition to Zn, Na was the only element decreasing in meat with *C. vulgaris* addition. Despite being an essential element, it is well established that high sodium intake increases blood pressure and the risk of cardiovascular disease. The WHO recommends reducing the salt intake to <5 g daily [58]. Although meat products are labeled as “high salt source” and not meat per se, the lower sodium amount in meat contributes to overall salt reduction in the diet. All breasts had a Na content lower than 0.12 g/100 g, hence a “low sodium/salt” claim can be made [19].

Few reports address the effect of cooking on the mineral content of chicken meat. The cooking process is important for safety and to enhance the sensory characteristics of meat [59]. However, thermal processing influences nutrient content in meat, including minerals [60]. In the present study, cooked breast meat showed higher Ca, Mg, P, Fe, Cu, Zn, and Mn contents and lower contents of Na and K than raw samples (Table 6). Findings from this study agree with previously reported results. Thermal processed meat from different animal species was characterized by a higher content of most minerals, with an exception of Na and K as compared to raw meat [60,61,62,63]. Tomovic et al. [64] reported that boiling pork loin increases the mineral content as the consequence of cooking loss, corresponding to a higher concentration of all elements. Purchas et al. [61] reported that although cooking increased mineral content of most elements, the mineral concentration in the dry matter of raw and cooked lean was similar except for sodium and potassium, which concentrations were lower in dry matter of cooked meat. Namely, divalent minerals bind to proteins which are not likely to decrease during cooking while sodium and potassium, monovalent elements, are released from meat into meat juice. Thus, in our study the relative increase of divalent elements in cooked meat is due to loss of meat juice during cooking and increase level of proteins in relative weight of the cooked sample compared to the raw one.

In the present study, considering the contribution of iron to the diet, an intake of 100 g of cooked breast meat would provide between 1.11 (control) and 1.69 (CV15%) mg/100 g, corresponding to 7.93–12.07% of the daily reference intakes (RDI) for adult women and men. Furthermore, the intake of 100 g of cooked breast would provide between 18.17-(C) and 19.43% (CV20%) K and between 34.28% (CV10%) and 35.98% (CV20%) P of daily reference intakes (RDI) for adults [19]. For P and K, chicken breast provides at least 15% of the reference daily intake, therefore, it could be labeled as “source of P and K” according to regulations EC No 1924/2006 and EU No 1169/2011. Furthermore, the amount of Cu in cooked broilers breast from CV 20% group is reaching 15% of RDI for this mineral.

**Figure 1:**
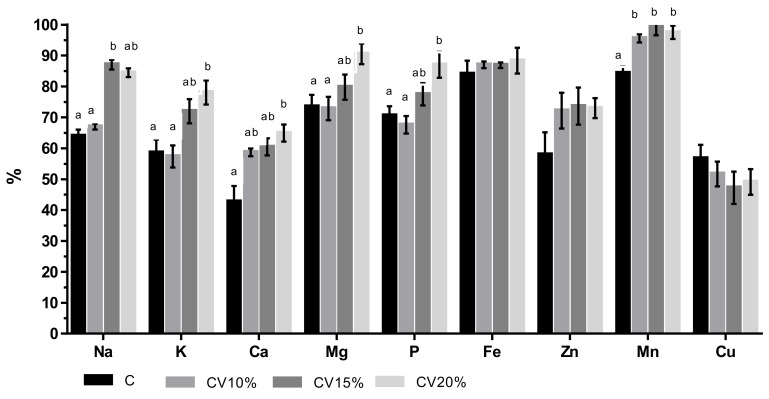
Mineral bioaccessibility in breast meat of broilers chickens fed different levels of *C. vulgaris* for 40 days. Data shown are mean ± SE, *n* = 6. ^a,b^ Different superscripts within a row indicate a significant difference (*p* < 0.05).

Although the determination of mineral content in raw and cooked meat certifies that the matrix is a good source or high in content of particular minerals, the contents of minerals available from food sources to humans is evaluated by their bioaccessibility. Minerals bioaccessibility, expressed as the percentage of mineral remaining in the digesta (fraction obtained after in vitro digestion using INFOGEST protocol), are presented in Figure 1.

An increasing trend of bioaccessibility was observed for most of the minerals in meat when *C. vulgaris* was included in broilers feed, with exception of Cu. There was no significant difference (*p* > 0.05) in Fe, Zn, and Cu bioaccessibility for the control breast meat and meat from broilers fed *C. vulgaris.* Results presented in Figure 1 indicate that the minerals are only partially available to be absorbed by the human organism. Higuera et al. [65] reported that the mineral bioaccessibility in lamb meat decreased by cooking process. Namely, denaturation of proteins can create additional binding sites between protein and metals, leading to metal trapping, thus reducing bioavailability. Additionally, some amino acids such as lysine, methionine, phenylalanine, histidine, and cystine have an affinity for ion [66]. The meat from this study was rich in those amino acids (Table 5), in particular lysine. Meat from the control group had higher amount of lysine and cystine compared to CV15% and CV20% groups which can partially be responsible for lower bioaccessibility of aforementioned elements in meat from control.

Mn bioaccessibility significantly increased by *C. vulgaris* addition (*p* ≤ 0.05). Mn was the mineral with the highest bioaccessibility, reaching up to 99%, with the highest bioaccessible fraction being achieved for breast meat from broilers fed 20% *C. vulgaris*. Foods from plant origin are the main source of Mn in human diet [67]. However, considering the present dietary role of meat and its average daily intake, Mn in meat is important since this essential mineral influences growth, enzymatic defense systems against oxidation and immune system [68,69]. Thus, any strategy leading to increase bioaccessibility would provide significant contributions to the amount of Mn ready to be absorbed. After Mn, the most bioaccessible minerals were Mg, P, and Fe. Furthermore, analysis of the effects of *C. vulgaris* dietary inclusion on meat mineral bioaccessibility showed that despite the dose-dependent increase observed, only the addition of 20% of this microalga significantly increased (*p* ≤ 0.05) K, Ca, Mg, and P bioaccessibility (*p* ≤ 0.05) compared to control. Although the inclusion of lower amounts of microalgae in broiler feed did not affect mineral bioaccessibility, it should be highlighted that cooked meat from groups fed *C. vulgaris* had higher concentration of minerals; thus, greater concentration of Fe, P, K, Ca, Mg, and Mn was available for absorption in the body, compared with the control meat. In addition, even if the amount of Zn was higher in control meat, the bioaccessible concentration in the digesta was similar to those in *C. vulgaris* groups, since the bioaccessibility (%) for this mineral was higher in meat from broilers fed this microalga.

It is difficult to compare values of in vitro digestion between studies due to different digestion protocols and enzymes used, different duration of digestion, sample preparation, and sample chemical compositions and method used to calculate the digestibility. In the case of mineral digestion, bile salts are an additional factor that has to be considered. While Rousseau et al. [70], found that Zn bioaccessibility was drastically reduced by adding bile salts in comparison to in vitro digestion where only enzymes were used, Muleya et al. [71] reported that not only bile salt but pancreatine can also interfere in Zn and Fe mineral binding and decrease bioaccessibility. Those authors showed that isotopic labeling of reagent iron and zinc is appropriate to accurately determine their bioaccessibility in grains and legumes based on a modified INFOGEST in vitro digestion method. Muleya et al. [71] also emphasized that saturated solutions of pancreatin and bile used in the INFOGEST in vitro digestion method, precipitate during centrifugation with the potential to adsorb metals into the residue. They suggest that traditional method for calculation of mineral bioaccessibility can either overestimate or underestimate Zn and Fe bioaccessibility leading to misinterpretation of results. No data on microalgae dietary regime on meat digestibility is available nor was the INFOGEST method used to evaluate mineral bioaccessibility from chicken meat; thus, it is not possible to compare our results with existing literature. Da Silva et al. [72] reported that Fe bioaccessibility was greater than 80% in baby food samples that contained chicken meat. Those findings are in line with those obtained in present study (84.03–88.33%; Figure 1). In an opposite trend, Menezes et al. [73] reported that the bioaccessibility in thermally processed chicken for Ca, Cu, Fe, Mg, and Zn ranged between 8 and 22%, 8 and 12%, 8 and 16%, 10 and 26%, and 8 and 16%, respectively. However, these authors used dialyzed fraction while in the present study soluble fraction was used. Câmara et al. [74], compared dialysis and solubility methods to determine bioaccessibility of minerals in school meals, including dishes containing chicken (chicken with vegetable stew and chicken in sauce). These authors found that the percentage of mineral solubility was higher for Fe, Zn, and Cu than dialyzed mineral percentage because the mineral may be bound to compounds of molecular sizes in excess of the pore size of the dialysis membrane. This could explain the difference between studies.

### 3.4. Proteins Recovery and Digestibility

**Figure 2:**
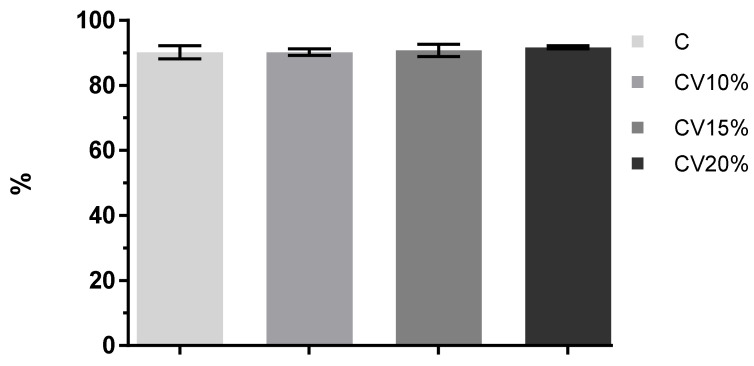
Digestibility of breast muscle of broilers chickens fed different levels of *C. vulgaris* for 40 days. Data shown are mean ± SE, *n* = 3.

Figure 2 presents the effect of dietary *C. vulgaris* inclusion on the digestibility (%) of cooked breast meat subjected to in vitro protein digestion. While inconsistency arises in research papers that refer to minerals bioaccessibility, reports agree that meat proteins have a high protein digestibility that can exceed 90% [75,76]. Furthermore, it has been reported that true fecal protein digestibility values for human adults from meat including poultry range between 90 and 99% [77]. Results from the present study confirm such data with total meat digestibility of approximately 90% regardless of dietary treatment.

Soluble and insoluble protein, as well as the total protein recovery values for the sum of soluble and insoluble protein, are presented in Table 7. All meat samples showed higher soluble protein and lower insoluble protein content. Meat from the control group had the least soluble protein (86.83%), followed by breast meat from CV10% (87.24%), CV15% (88.24%), and CV20% group (90.08%). The total protein recovery ranged from 96.7 to 98.4%. The high recovery rates confirm the reliability of methodology despite the complexity of the food matrix and protocol used and agree with results reported by Rieder et al. [21], who obtained a similar protein recovery rate for chicken mince after the intestinal phase (98.7%) using the INFOGEST protocol.

These authors reported a similar, but lower, value for soluble protein content (83.3%), which could be due to the use of raw meat, while in our study cooked meat was used for digestion. How cooking influences protein digestion depends, among other factors, on temperature. Processing meat at moderate temperatures leads to the unfolding of buried residues through denaturation, hence allowing digestive enzymes access to protein cleavage sites and improving protein digestibility [76,78,79]. Contrary, processing at high temperatures causes protein oxidation, aggregation, and gel formation and decreases protein digestion due to lower proteolytic susceptibility [76,79]. Bax et al. [78] reported an increase in meat digestibility after meat being exposed to 70 °C cooking temperature, while cooking temperatures of 100 °C and 140 °C, decreased digestibility by 40% compared to raw meat. In the present study, the breast meat was cooked at lower temperatures (80 °C) which could be the reason for increased soluble protein content. As mentioned previously, protein oxidation due to cooking decreases digestibility. Mechanisms of protein oxidation vary between amino acids. Lysine and proline have a tendency for the formation of carbonyls on side chains while some amino acids including cysteine formed disulphide cross-links [80]. In the present study, breast meat of the control group had a higher % of lysine and cysteine in total amino acid composition compared to the groups with 15 and 20% *C. vulgaris* inclusion, which could be the reason for greater quantities of soluble proteins obtained from meat of broilers fed *C. vulgaris*.

## 4. Conclusions

This study demonstrated that partial replacement of soybean meal with *C. vulgaris* in broilers diet imparts a significant influence on meat chemical composition. The inclusion of this microalga in the broiler diet, increased protein and ash, and decreased fat content in the broiler breast meat. The mineral content was affected in a dose-dependent manner. The highest amount of minerals was reported when 20% of *C. vulgaris* was included in broiler feed, aside from Na and Zn, which decreased by *C. vulgaris* addition. The higher iron content, in raw and cooked meat from broilers fed *C. vulgaris* could contribute significantly to the daily requirements. Furthermore, cooking increased the relative mineral content as a consequence of cooking loss, with the exception of Na and K. Moreover, as shown by the in vitro INFOGEST model, breast meat is an excellent source of digestible proteins. Additionally, *C. vulgaris* inclusion in broilers feed increased mineral bioaccessibility probably partially due to the modification of amino acid composition. The microalga inclusion in broilers’ diet did not have a significant influence on Fe, Zn, and Cu bioaccessibility in meat. 

Overall, the results from this study confirm that *C. vulgaris* can be used as a valuable source of protein in broiler diets while improving nutritional composition of meat. However, given that this is the first report on the impact of microalgae in animal feed on the digestibility and minerals bioaccessibility in meat, this topic requires further research to fully understand the mechanisms behind increased digestibility.

## Figures and Tables

**Table 1:** Chemical composition of *C. vulgaris* (DM basis).

Ingredients (%)	
Dry matter	93.1
Ash	12.7
Crude Protein	46
Crude fat	9.4
Gross energy:	4586 cal/g

**Table 2:** Ingredient composition and nutrient levels of experimental diets (%).

	Dietary Treatments							
Item	Starter				Grower			
	C	CV10%	CV15%	CV20%	C	CV10%	CV15%	CV20%
Ingredients (%)								
Premix	0.4	0.4	0.4	0.4	0.4	0.4	0.4	0.4
Salt	0.35	0.35	0.35	0.35	0.35	0.35	0.35	0.35
Corn	43.00	45.36	46.13	46.51	50.18	52.00	52.71	53.42
Soybean Meal	48.00	39.00	34.00	29.2	41.5	31.56	26.61	21.7
*C. vulgaris* powder	0	10	15	20	0	10	15	20
Sunflower Oil	3.5	1.64	0.75	0.00	4.80	2.89	1.99	1.10
DL-Methionine	0.13	0.18	0.21	0.24	0.11	0.16	0.19	0.21
L-Lysine	0.00	0.13	0.30	0.47	0.00	0.15	0.31	0.47
Calcium Carbonate	1.2	1.5	1.65	1.8	1.06	1.37	1.53	1.67
Dicalcium Phosphate	1.9	1.44	1.22	1.03	1.6	1.13	0.91	0.68

Dietary treatments: C: control, corn-soybean basal diet; CV10%, basal diet plus 10% *C. vulgaris*; CV15%, basal diet plus 15% *C. vulgaris*; CV20%, basal diet plus 20% *C. vulgaris.* Premix provided the following per kilogram of diet: pantothenic acid 10 mg, vitamin D_3_ 2400 IU, cyanocobalamin 0.02 mg, folic acid 1 mg, vitamin K_3_ 2 mg, nicotinic acid 25 mg, vitamin B_6_ 2 mg, vitamin A 10,000 UI, vitamin B_1_ 2 mg, vitamin E 30 mg, vitamin B_2_ 4 mg, Cu 8 mg, Fe 50 mg, I 0.7 mg, Mn 60 mg, Se 0.18 mg, Zn 40 mg.

**Table 3:** Amino acids profiles of experimental diets (g/100 g).

Amino Acids	Dietary Treatment			
	C	CV10%	CV15%	CV20%
Essential amino acids:				
Histidine	0.72	0.74	0.77	0.80
Isoleucine	0.80	0.87	0.87	0.91
Leucine	1.48	1.49	1.50	1.50
Lysine	0.74	0.85	1.05	1.11
Methionine	0.30	0.30	0.31	0.33
Phenylalanine	0.69	0.85	0.93	1.02
Threonine	0.75	0.76	0.81	0.85
Tryptophan	0.35	0.36	0.37	0.38
Valine	1.04	1.10	1.11	1.12
Nonessential amino acids:				
Arginine	1.62	1.65	1.67	1.69
Alanine	1.21	1.26	1.28	1.29
Aspartic acid	4.29	4.11	4.19	4.29
Cysteine	0.25	0.29	0.29	0.30
Glutamic acid	3.08	3.10	3.13	3.18
Glycine	0.81	0.82	0.88	0.90
Proline	1.25	1.22	1.26	1.26
Serine	0.97	0.94	0.97	0.99
Tyrosine	0.70	0.72	0.77	0.78
Total amino acid/Protein content	21.05	21.40	22.19	22.7

**Table 4:** Chemical composition and cholesterol in breast muscle of broilers chickens fed different levels of *C. vulgaris* for 40 days.

Parameter	Dietary Treatment				SEM	*p* Value
	C	CV10%	CV15%	CV20%		
Moisture (%)	71.4	72.23	70.93	70.90	1.006	0.1620
Proteins (%)	24.4 ^a^	25.56 ^ab^	27.1 ^b^	26.89 ^b^	1.116	0.0083
Fat (%)	3.40 ^a^	1.95 ^b^	0.92 ^c^	1.22 ^bc^	0.786	0.0006
Ash (%)	0.82 ^a^	1.04 ^b^	1.05 ^b^	0.99 ^b^	0.053	0.00002
Cholesterol (mg/100 g)	37.5	40.67	43.00	39.00	0.059	0.597
Energy (kcal/100 g)	128.1	119.79	116.68	118.54	7.905	0.139

^a,b,c^ Different superscripts within a row indicate a significant difference (*p* < 0.05).

**Table 5:** Profiles of amino acids in breast muscle of broilers chickens fed different levels of *C. vulgaris* for 40 days.

Amino Acids (% of Total Amino Acids)	Dietary Treatment				SEM	*p* Value
	C	CV10%	CV15%	CV20%		
Essential amino acids:						
Histidine	4.30	4.26	4.15	4.22	0.241	0.8294
Isoleucine	2.95	2.84	3.12	3.16	0.412	0.6654
Leucine	6.10	5.86	5.94	6.04	0.318	0.7388
Lysine	14.69 ^a^	14.14 ^ab^	13.58 ^bc^	13.11 ^c^	0.516	0.0021
Methionine	2.69	2.82	2.97	2.88	0.161	0.1209
Phenylalanine	3.51	3.56	3.47	3.53	0.052	0.1279
Threonine	4.71 ^a^	5.10 ^b^	5.27 ^bc^	5.54 ^c^	0.143	<0.0001
Valine	3.43	3.42	3.69	3.64	0.433	0.7438
Nonessential amino acids:						
Alanine	6.28	6.73	6.60	6.94	0.372	0.1165
Arginine	11.40 ^a^	12.13 ^b^	12.19 ^b^	12.33 ^b^	0.273	0.0005
Aspartic acid	8.26	8.32	8.25	8.22	0.140	0.8188
Cysteine	2.75 ^a^	2.19 ^ab^	2.30 ^ab^	1.81 ^b^	0.442	0.0499
Glutamic acid	13.18	13.20	12.99	12.68	0.746	0.1561
Glycine	4.00	4.14	4.06	4.31	0.212	0.2206
Proline	5.10	4.89	4.76	5.06	0.268	0.2796
Serine	3.18	3.19	3.26	3.19	0.146	0.8728
Tyrosine	3.49	3.22	3.42	3.36	0.263	0.5333

^a,b,c^ Different superscripts within a row indicate a significant difference (*p* < 0.05).

**Table 6:** Mineral composition (mg/100 g) of raw and cooked breast muscle of broilers chickens fed different levels of *C. vulgaris* for 40 days.

Sample	Minerals								
	Na	K	Ca	Mg	P	Fe	Cu	Zn	Mn
Raw breast meat									
Control	69.9 ^a^	357.6 ^a^	4.70 ^a^	28.3 ^a^	223.7 ^a^	1.09 ^a^	0.053	1.26	0.01
CV10%	59.7 ^ab^	370.1 ^ab^	4.7 ^a^	30.4 ^ab^	231.4 ^ab^	1.19 ^ac^	0.065	1.17	0.02
Increase/Decrease * (%)	−14.6	+3.5	/	+7.5	+3.4	+8.7	+22.6	−7.3	+100
CV15%	54.5 ^b^	398.9 ^ab^	6.7 ^b^	33.1 ^b^	254.0 ^b^	1.58 ^b^	0.065	1.03	0.023
Increase/Decrease * (%)	−22.0	+11.5	+41.7	+8.9	+13.5	+45.1	+22.6	−18.7	+133.3
CV20%	58.8 ^ab^	402.6 ^b^	6.4 ^b^	31.6 ^ab^	249.8 ^ab^	1.45 ^bc^	0.065	1.02	0.025
Increase/Decrease * (%)	−15.9	+12.6	+35.3	+11.6	+11.6	+33.2	+22.6	−19.9	+150
SEM	9.899	21.790	1.184	2.032	13.655	0.246	0.011	0.263	0.010
*p* value	0.024	0.021	0.001	0.022	0.014	0.001	0.333	0.493	0.210
Cooked breast meat									
Control	65.1 ^a^	363.5	4.8 ^a^	30.8 ^a^	244	1.11 ^a^	0.126 ^a^	1.64 ^a^	0.024 ^a^
CV10%	62.3 ^a^	361.3	5.8 ^b^	31.22 ^ab^	240	1.27 ^ab^	0.131 ^ab^	1.21 ^b^	0.026 ^ab^
Increase/Decrease * (%)	−4.4	/	+20.4	+1.36	−1.6	+14.2	+3.97	−26.0	+8.3
CV15%	50.3 ^ab^	388	6.2 ^bc^	34.93 ^bc^	258.9	1.60 ^ab^	0.149 ^b^	1.05 ^b^	0.025 ^ab^
Increase/Decrease * (%)	−22.8	+6.7	+29.2	+13.4	+6.1	+43.5	+18.25	−35.8	+4.2
CV20%	47.6 ^b^	388.5	7.2 ^c^	35.8 ^c^	251.9	1.69 ^b^	0.150 ^b^	1.03 ^b^	0.029 ^b^
Increase/Decrease * (%)	−26.9	+6.9	+49.7	+16.3	+3.2	+51.8	+18.25	−36.9	+20.8
SEM	6.329	20.45	0.311	1.929	13.99	0.255	0.011	0.127	0.002
*p* value	0.002	0.115	<0.00001	0.002	0.259	0.014	0.008	<0.0001	0.037

* Increase/decrease compared to control group; ^a,b,c^ Different superscripts within a row indicate a significant difference (*p* < 0.05).

**Table 7:** Insoluble protein and soluble protein after in vitro digestion of breast muscle of broilers chickens fed different levels of *C. vulgaris* for 40 days. Data shown are mean ± SE.

Breast Meat	Protein Recovery [%] ± SE		
	Total protein	Soluble protein	Insoluble protein
Control	96.66 ± 1.40	86.83 ± 1.56	9.83 ± 1.16
CV10%	97.07 ± 1.19	87.24 ± 1.62	9.83 ± 0.58
CV15%	97.48 ± 1.78	88.24 ± 1.92	9.25 ± 1.10
CV20%	98.39 ± 1.58	90.08 ± 1.70	8.31 ± 0.28

## Data Availability

Data is contained within the article.
